# The contributing role of CCR5 in dementia

**DOI:** 10.3389/fneur.2025.1545302

**Published:** 2025-07-09

**Authors:** Tong Zheng, Meiping Ye, Pingyu Zhou

**Affiliations:** ^1^STD Institute, Shanghai Skin Disease Hospital, Tongji University School of Medicine, Shanghai, China; ^2^Department of Dermatology, Xinhua Hospital, Shanghai Jiaotong University School of Medicine, Shanghai, China

**Keywords:** CCR5, dementia, cognitive decline, learning and memory, synaptic plasticity

## Abstract

Dementia is a syndrome of impaired brain function in which cognitive functions such as memory, language, attention, direction, and judgment are impaired, affecting or interfering with daily functioning. As dementia becomes more widespread, it is crucial to investigate the underlying mechanisms that contribute to cognitive decline. C-C chemokine receptor 5 (CCR5) has been extensively researched for its role in immune responses and function as a co-receptor in HIV infection. Current research indicates that CCR5, which acts as a regulator of synaptic plasticity, is involved in modulating various forms of learning and memory. Most studies suggest that CCR5 generally has a detrimental effect on diseases associated with dementia. This review seeks to deliver an extensive analysis of CCR5’s role in cognitive processes by summarizing existing literature from both animal and human studies. It will cover the involvement of CCR5 in standard learning and memory functions, as well as in various types of dementia. The review will specifically address conditions such as HIV-related neurocognitive impairment (HAND), Alzheimer’s disease (AD), stroke, vascular dementia, multiple sclerosis (MS), frontotemporal dementia (FTD), dementia with Lewy bodies (DLB), and Parkinson’s disease with dementia (PDD). Based on the fact that CCR5 plays a contributing role in many diseases that cause dementia, this review also proposed CCR5 inhibition as a possible target for alleviating and ameliorating dementia.

## 1 Introduction

Dementia encompasses a significant cognitive decline that disrupts daily independence and is more accurately described as a syndrome rather than a single disease. It results from various primary neurologic, neuropsychiatric, and medical conditions, often involving multiple contributing diseases (1). With more than 55 million individuals impacted worldwide and nearly 10 million new cases emerging each year, the global prevalence of dementia is projected to increase to 152.8 million by 2050. In China alone, approximately 15 million people over 60 years live with dementia, representing a quarter of the global total (2).

Advancing age, genetic factors, systemic vascular diseases, and infections are key risk factors for dementia (3–5). Dementia is often categorized into neurodegenerative and non-neurodegenerative diseases (Table 1), with neurodegenerative conditions being the predominant cause. Mixed dementia, involving different elements, is also common (1). Proinflammatory biomarkers correlate with the extent of cognitive deterioration. The hippocampus, crucial for memory and learning, is especially vulnerable to neural impairments due to inflammation, owing to its elevated inflammatory marker expression (6). Among these markers are Interleukin-1*α* (IL-1α), Interleukin-1β (IL-1β), Interleukin-6 (IL-6), tumor necrosis factor-α (TNFα), and CCL2, contributing to microglial activation, synaptic irregularities, cognitive decline, and hindered adult neurogenesis (7, 8).

**Table 1:** Classification of causes of dementia.

Causes of neurodegenerative dementia	Causes of non-neurodegenerative dementia
Alzheimer’s disease Dementia with Lewy bodies Vascular dementia Frontotemporal lobar degeneration Parkinson’s disease	Vitamin deficiencies (e.g., B12, thiamine) Hypothyroidism Normal pressure hydrocephalus Chronic alcohol abuse Chemotherapy-related cognitive dysfunction Infections (e.g., human immunodeficiency virus, neurosyphilis) Intracranial masses (e.g., subdural hematomas, brain tumors) Stroke and traumatic brain injury Psychiatric illness

Chemokine receptors are increasingly recognized for their functions in cognitive function (9). CCR5, in particular, has been studied for its involvement in various neuroinflammatory processes and its impact on cognition. These studies have focused on diseases such as HIV-related neurocognitive impairment (HAND) (Supplementary Table 1), Alzheimer’s disease (AD) (Supplementary Table 2), Stroke (Supplementary Table 3), multiple sclerosis (MS) (Supplementary Table 4), Vascular dementia (10), Frontotemporal Dementia (FTD) (11), Dementia with Lewy Bodies (DLB) (12), and Parkinson’s disease with dementia (PDD) (12). Most studies on CCR5 have indicated that dementia is associated with elevated expression of CCR5 or its ligands. These findings propose that either inactivating CCR5 or causing its deficiency might diminish inflammation and improve cognitive function. However, two studies have posited that CCR5 deletion may exacerbate memory dysfunction and enhance neuronal death in AD and stroke (13, 14).

## 2 CCR5 and its role in learning and memory

**Figure 1:**
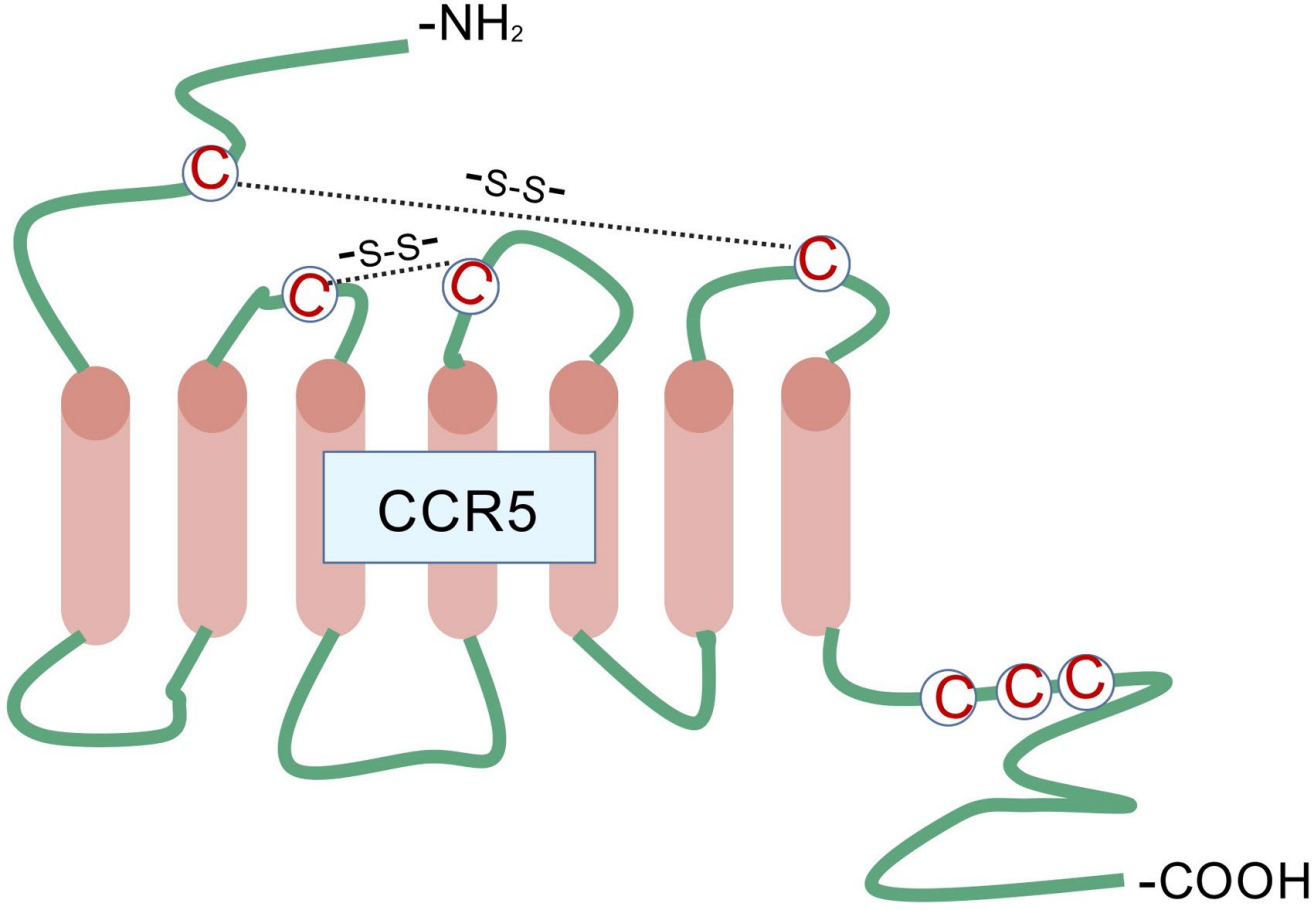
CCR5 belongs to the family of G protein-coupled receptors characterized by seven transmembrane domains, along with three extracellular and three intracellular loops. The extracellular N-terminus, positioned on the outer surface of the plasma membrane, serves as the principal site for chemokine ligand recognition and initial interaction. Extracellular loop regions cooperate with both the N-terminal domain and transmembrane segments to maintain the stability of the ligand-receptor complex, while also mediating structural rearrangements following ligand engagement to facilitate signal propagation across the membrane. The seven transmembrane α-helical bundles undergo spatial reorientation upon ligand binding, resulting in receptor activation through transition from a resting to an activated conformation - an essential mechanistic event in signal transduction. The intracellular loop structures function as molecular relays, conveying structural alterations from the transmembrane core to downstream intracellular signaling molecules. These loops additionally contain phosphorylation motifs targeted by GPCR kinases, playing pivotal roles in processes such as receptor desensitization and subsequent internalization. This figure was created with BioGDP.com.

CCR5 is a G protein-coupled receptor (GPCR) with seven transmembrane domains (Figure 1), and its gene is situated on chromosome 3p21. Because of its important function in the immune system, CCR5 expression is observed in various immune cells (15). In the central nervous system (CNS), CCR5 is highly enriched in many brain regions, including the CA1 region of the hippocampus and cortex (16). The hippocampus and cortex are essential for acquiring, consolidating, and retrieving episodic and spatial memories, which contribute to cognitive development. Compared with astrocytes and neurons, CCR5 is expressed more in microglia in the CNS (17). CCR5 is thought to be involved in the immunological processes and inflammation within the CNS. However, some evidence shows that CCR5 activation may also influence brain functions beyond immune responses, potentially affecting brain development and neuronal transmission. CCR5 interacts with several ligands, including CCL3, CCL4, CCL5, CCL8, CCL11, CCL14a, and CCL16 (18).

Numerous studies have investigated CCR5’s role in learning and memory (Table 2). It is considered a significant inhibitor of neuronal plasticity in the hippocampus and cortex, thereby diminishing learning and memory capabilities (16). CCR5 affects these cognitive functions by modulating the mitogen-activated protein kinases (MAPKs) (19, 20) and cAMP-responsive element-binding protein (CREB) (19, 21, 22) signaling pathways, which are crucial for hippocampal learning, memory, and cortical plasticity. Disruption of CCR5 function leads to increased levels of MAPK and CREB during learning, which boosts synaptic plasticity, and long-term potentiation (LTP) (23), and improves hippocampal learning and memory. Conversely, overexpression of CCR5 results in deficits in these cognitive areas. While CCR5 knockout does not alter MAPK or CREB signaling under baseline conditions, it increases their levels after training. This enhanced signaling correlates with improved learning and memory. In line with its effects on MAPK/CREB signaling, CCR5 knockout also enhances LTP in the hippocampal CA1 region and improves performance in diverse recognition tasks (16). Inhibition of CCR5 with maraviroc, similar to genetic CCR5 ablation, also enhances contextual memory in rats following fear conditioning training (24). In middle-aged mice, the administration of maraviroc to block CCR5 alleviated deficits related to memory linkage (21). This finding aligns with the notion that reducing CCR5 levels can enhance learning and memory. In contrast, transgenic mice with CCR5 overexpressed in excitatory neurons exhibited impaired learning and memory, indicating that CCR5 serves an inhibitory function in neuronal plasticity and memory processes.

**Table 2:** Studies of CCR5 in learning and memory.

Methods	Model	Main findings	Ref.
Ccr5 knockout mice, Ccl5 knockout mice, and aged mice using maraviroc were tested for contextual memory connectivity.	Mouse	Increasing neuronal CCR5 activity specifically impaired contextual memory. Both Ccr5 KO and Ccl5 KO showed an extended linking window. The administration of maraviroc, a CCR5 blocker, improved memory-related deficits in middle-aged mice.	Shen et al. (21)
The retention of fear memory was determined 24 h after the fear memory consolidation training course. The levels of these chemokines (CCR5 / CCL5) and IL-6 were measured in the hippocampus and prefrontal cortex of chronically stressed rats.	Rat	Chronic stress and contextual fear conditioning lead to increased expression of CCR5 and CCL5. Pre-treatment with maraviroc before contextual fear conditioning improves memory consolidation.	Merino et al. (24)
Ccr5 knockout mice, Ccr5+/ -mice, and CCR5-overexpressing transgenic mice were examined for neuroplasticity, learning, and memory.	Mouse	CCR5 knockdown strengths MAPK or CREB signaling and long-term potentiation (LTP), which can boost experience-dependent plasticity and memory. Neuronal CCR5 overexpression led to deficits in learning and memory.	Zhou et al. (16)
The effects of CCL3 on hippocampal synaptic transmission, plasticity, and spatial memory were assessed.	Mouse	CCL3 activation of CCR5 impairs hippocampal synaptic transmission, plasticity, and LTP, which negatively affects memory and cognitive behaviors. Maraviroc, a CCR5 antagonist, can prevent these adverse effects.	Marciniak et al. (25)

Similarly, CCR5 activation by its ligand CCL3 negatively impacts the performance of Y-maze tasks. These effects can be counteracted by maraviroc, a CCR5 antagonist. Notably, maraviroc also completely prevents CCL3-induced impairments in long-term potentiation (LTP). Importantly, maraviroc does not affect plasticity in the absence of CCL3 activation, indicating that CCR5’s tonic activity alone does not influence synaptic plasticity (25). During the contextual memory linking test, the CCL5 group exhibited diminished contextual memory linking, reflecting that increased CCR5 activity impairs this process. Likewise, CCL5 knockout (Ccl5−/−) mice, like CCR5 knockout (Ccr5−/−) mice, displayed a prolonged linking window, underscoring the importance of CCL5 in regulating CCR5’s role in memory linking. An age-related rise in CCL5–CCR5 expression results in memory-linking impairments in aged mice, which can be reversed by maraviroc, suggesting significant potential for clinical applications (21).

## 3 CCR5 in HIV-associated neurocognitive disorder (HAND)

HIV-associated neurocognitive disorder (HAND) is a comprehensive syndrome of neurological deficits observed in individuals with HIV, manifesting in various stages of cognitive, behavioral, and motor impairments (26). These may include difficulties with complex tasks, delayed speech, diminished initiative, reduced fine motor skills and speeds, unsteady gait, and deficits in learning and memory. A recent meta-analysis of 225 studies reveals that approximately 40% of people living with HIV experience cognitive impairment (27).

CCR5 serves as a coreceptor for HIV entry into host cells after the gp120 binds to the CD4 receptor (28). HIV-infected cells, cross the blood–brain barrier, leading to viral replication in microglia and macrophages and subsequent brain infection (29). Additionally, astrocytes and neurons may also act as target cells for HIV (30). Up-regulation of CCR5 has been observed in brain samples from AIDS patients (31), as well as in microglia, astrocytes, and neurons exposed to HIV proteins (32). Some evidence shows that CCR5 may worsen cognitive impairments linked to HIV infection (Supplementary Table 1), and the cognitive performance of HIV patients has been improved to varying degrees after the use of pharmacological inhibitors of CCR5 (33–35).

CCR5 may contribute to HAND by promoting the release of neurotoxic mediators through activated glial cells. This process involves CCR5-mediated secretion of cytokines like TNF-*α*, IL-1β, and IL-6, observed in astrocytes stimulated with the HIV-1 protein gp120 (36). The interaction of HIV-1/gp120 with CCR5 on microglia leads to their activation and subsequent toxin production, which appears crucial for causing significant neurodegeneration. Kaul et al. demonstrated that gp120-induced neurotoxicity was significantly decreased when microglia were either removed or inactivated in mixed neuronal/glial cerebrocortical cultures (37). This finding highlights the crucial role of microglial receptors CCR5 in mediating neurotoxicity. The data suggest that microglia are important for the neurotoxic effects of gp120, indicating that these cells, through their interaction with HIV-1 coreceptors, are central to the neurodegenerative processes observed in HAND.

**Figure 2:**
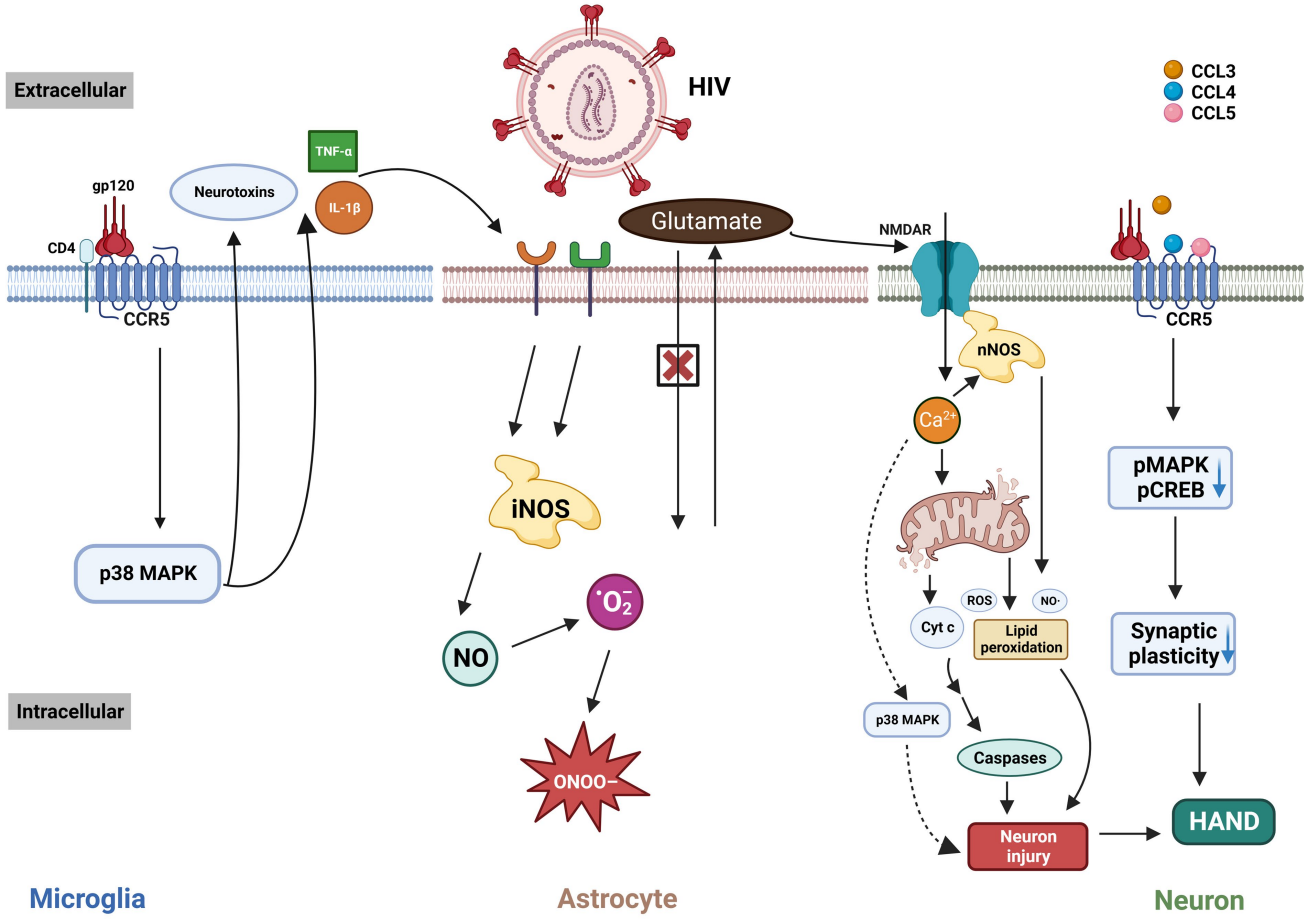
The interaction of HIV/gp120 with CCR5 and CD4 triggers microglial activation, leading to a signaling cascade involving p38 MAPK that leads to the release of neurotoxic substances and inflammatory cytokines like TNF-α and IL-1β. These cytokines activate microglia, astrocytes, and other cells, exacerbating brain injury. This process impairs glutamate uptake and increases neurotoxic nitric NO production. Elevated glutamate leads to NMDAR overstimulation, excessive Ca²^+^ influx, and subsequent neuronal damage through mitochondrial dysfunction, free-radical generation, and apoptosis. The binding of HIV-related ligands to CCR5 also reduces pMAPK and pCREB levels, impairing synaptic plasticity and worsening cognitive deficits in HAND. MAPK: mitogen-activated protein kinases. iNOS: inducible nitric oxide synthase. ONOO-: peroxynitrite. NO: nitric oxide. ·O^2-^: superoxide anion radical. NMDAR: N-methyl-D-aspartic acid receptor. nNOS: neuronal nitric oxide synthase. ROS: reactive oxygen species. Cyt c: Cytochrome c. CREB: cAMP-responsive element-binding protein. HAND: HIV-associated neurocognitive disorder. This figure was created with BioRender.com.

HIV/gp120 binds to CCR5 and CD4, activating microglia and macrophages via the p38MAPK signaling pathway (Figure 2). This activation leads to the release of neurotoxic substances such as glutamate (38) and inflammatory cytokines (TNF-*α*, IL-1β), which further stimulate adjacent glial cells like astrocytes (39). These astrocytes release more inflammatory cytokines (40) and nitric oxide (NO), forming peroxynitrite (ONOO-), which is neurotoxic (41). Glutamate release from microglia/macrophages causes excessive activation of the N-methyl-D-aspartic acid receptor (NMDAR), leading to harmful intracellular signals, and eventually, neurons may undergo necrosis or apoptosis. Additionally, CCR5 knockout or CCR5 antagonists in HIV mouse models reduce microglial activation and accumulation, preventing gp120-induced spatial memory deficits (42).

Another potential pathway through which CCR5 contributes to HIV-related cognitive impairment involves the direct activation of CCR5 on neurons by gp120 (16) (Figure 2). This activation reduces MAPK/CREB signaling, which in turn leads to deficits in LTP, affecting synaptic plasticity and resulting in cognitive impairments. Similar to the endogenous CCR5 ligands (43), HIV gp120 V3 peptides can bond to and activate CCR5. Administration of the V3 peptide before fear conditioning resulted in a contextual memory deficit in wild-type mice, whereas Ccr5+/− and Ccr5 −/− mice showed a marked improvement in memory function. The expression of MAPK phosphorylation (pMAPK) in the CA1 subregion of the dorsal hippocampus was decreased after learning, indicating that the V3 peptide may affect memory by interfering with pMAPK in the hippocampal CA1 region.

Although astrocytes do not normally produce intact virions under standard conditions, they can make and release nonstructural proteins (44) such as Tat, an HIV transcription factor, when infected with HIV (45). These proteins stimulate inflammation and neuronal damage. Unlike gp120, which binds directly to CCR5, Tat does not interact directly with CCR5. In mixed neuronal/glial cocultures consisting of astrocytes, microglia, and other glial cells, CCR5 removal from glial cells reduced neuronal death treated with HIV Tat protein in combination with morphine. This protective outcome associated with CCR5 loss was observed with pretreatment with the CCR5 antagonist maraviroc (46, 47). Given that these experiments were performed in HIV-1 Tat transgenic mice, the findings demonstrate that CCR5 can influence HAND independent of direct interactions with HIV/gp120.

## 4 CCR5 in Alzheimer’s disease (AD)

Alzheimer’s disease (AD) is the most common type of dementia (48), corresponding to about 65% of all dementia cases. AD commonly presents with a gradual decline in episodic memory and cognitive function, eventually contributing to impairments in language and visuospatial skills (49). The rate at which dementia symptoms progress from mild to moderate and then to severe can vary widely among individuals. Extracellular plaques of insoluble *β*-amyloid peptide (Aβ), neurofibrillary tangles (NFT) of P-tau in neuronal cytoplasm, and destruction of neurons, called neurodegeneration are the hallmarks of AD (50, 51).

Many studies have documented elevated CCR5 in both AD patients and animal models of AD (Supplementary Table 2), such as mice and rats. This heightened CCR5 expression has been observed in brain samples from individuals with AD (52). Moreover, CCR5 ligands, specifically CCL3 and CCL4, are also upregulated in Aβ-stimulated microglia in AD brains and APP/PS1 mouse models of the disease (53–55). This suggests that not only is CCR5 expression increased in AD, but the elevated levels of its ligands may further amplify CCR5 activity, potentially contributing to the progression of AD. This relationship highlights the possible involvement of CCR5 in the pathophysiology of AD and points to potential therapeutic targets for managing or slowing the disease.

**Figure 3:**
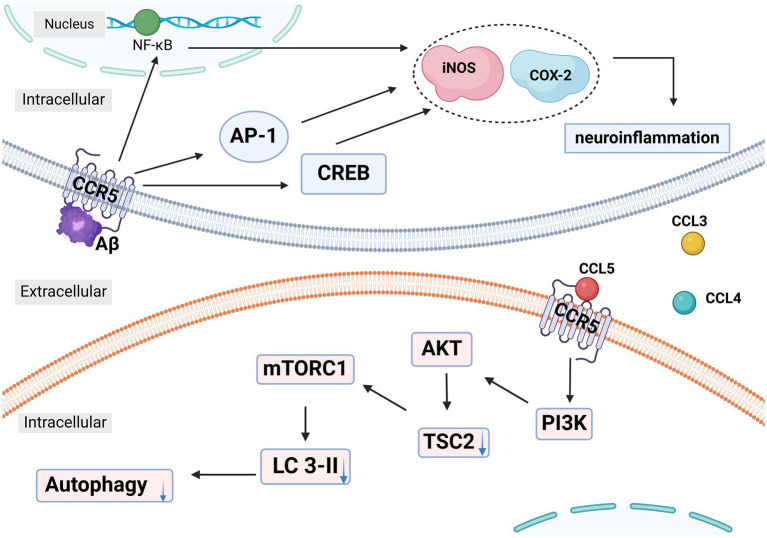
In Alzheimer’s disease (AD), the activation of CCR5 on microglia and astrocytes by Aβ leads to the increased expression of NF-κB, AP-1, and CREB, which enhances the production of neuroinflammatory mediators like COX-2 and iNOS. This neuroinflammation is further exacerbated by the secretion of CCL3, CCL4, and CCL5 by microglia. These chemokines activate CCR5, which in turn increases mTORC1 activity via the PI3K-AKT-TSC2 pathway. This disruption of autophagy contributes to the accumulation of tau and Aβ, aggravating neurodegenerative processes. NF-κB: nuclear factor-κB. AP-1: activator protein-1. CREB: cAMP-responsive element-binding protein. iNOS: inducible nitric oxide synthase. COX-2: cyclooxygenase-2. LC3: light chain. PI3K: Phosphatidylinositol 3-kinase. AKT: protein kinase B. TSC2: Tuberous Sclerosis Complex-2. This figure was created with BioRender.com.

In Passos et al.’s study (56), AD was assessed by evaluating mice’s ability to learn and recall spatial information using the water maze paradigm. CCR5−/− mice showed an increased time spent in the correct quadrant during testing, indicating less cognitive impairment from A*β*1-40 injection. The study also noted a marked increase in activated astrocytes and microglia in response to Aβ. Since glial cell activation is an early AD pathology and often triggered by Aβ accumulation, the study attributed the cognitive improvements in CCR5 knockout mice to reduced glial activation and β-amyloid accumulation. Passos et al. found reduced levels of inducible nitric oxide synthase (iNOS) and cyclooxygenase-2 (COX-2) in the hippocampus of CCR5−/− mice. Their previous work highlighted CREB, nuclear factor-κB (NF-κB), and activator protein-1 (AP-1) as crucial regulators of COX-2 and iNOS expression triggered by Aβ in the mouse hippocampus. The study indicates that the reduced expression of these enzymes in CCR5-deficient mice is linked to diminished activation of AP-1, NF-κB, and CREB. Consistent with the effect of CCR5 gene knock-out, the CCR5 antagonist DAPTA decreased microglia and astrocyte activation in the hippocampus, along with reducing the number of cells expressing NF-κB protein, a key player in pro-inflammatory cytokine signaling pathways, in a neuroinflammatory rat model of AD (Figure 3) (57). These findings suggest that DAPTA might alleviate crucial factors of neuroinflammation associated with AD.

Festa et al. found elevated CCR5 levels in neurons of rTg4510 and PS19 tauopathy mouse models (58, 59). They also observed increased concentrations of CCL3/4/5 in the PS19 mice. Elevated Mechanistic Target of Rapamycin Complex 1 (mTORC1) signaling and decreased light chain (LC3)-II levels in these mice indicated impaired autophagy, as mTORC1 inhibits this process. CCR5 depletion and inhibition with maraviroc normalized mTORC1 signaling and LC3-II levels, and improved cognitive function in PS19 mice (58, 59). Their study demonstrated that CCL3/4/5 secreted by activated microglia was responsible for inhibiting autophagy. Immunodepletion of these chemokines or suppression of mTORC1 activity prevented the reduction in LC3-II levels. CCL3/4/5 did not activate mTORC1 in CCR5-KO neurons, suggesting that these chemokines signal through CCR5 to affect mTORC1 activity (59). These experiments showed that CCL3/4/5 secreted by activated microglia, activated CCR5 on neurons, caused mTORC 1 activation, and decreased LC3-II level, thus inhibiting neuronal autophagy and ultimately leading to abnormal aggregation of tau protein (Figure 3).

While many studies link increased CCR5 expression with AD pathology and suggest that CCR5 inhibition could improve cognitive function (56–60), there is evidence that reducing CCR5 might increase Aβ deposition and memory impairment (14). This paradox is thought to occur because CCR5 deletion can upregulate CCR2, leading to activated astrocytes, increased Aβ production, and hippocampal cellular apoptosis, potentially worsening the disease (14, 55, 61).

## 5 CCR5 in stroke, traumatic brain injury (TBI) and vascular dementia

Stroke and traumatic brain injury (TBI) are major contributors to adult disability due to their impact on neurological recovery (62). Cognitive impairment is notably prevalent following a stroke. Statistics indicate that approximately 10% of individuals who experience a first stroke will develop dementia (63). This risk increases with recurrent strokes, with about one-third of people suffering a recurrent stroke also developing dementia. Additionally, milder cognitive deficits, including memory impairments, affect around 40% of stroke survivors (63). These cognitive issues can markedly affect daily functioning and quality of life, underscoring the need for effective rehabilitation and management strategies.

A study observed that deleting CCR5 led to increased neuronal death and larger infarcts in mice with induced cerebral ischemia (Supplementary Table 3). Despite this, there were no notable differences in astrocytes and microglia between wild-type and CCR5-deficient mice under occlusion (13). Following transient cerebral ischemia caused by bilateral common carotid artery occlusion (BCCAo), CCR5-deficient mice displayed reduced necrotic cavity areas and fewer ischemic neurons. Furthermore, these CCR5−/− mice exhibited elevated levels of the neurotrophic factor BDNF compared to wild-type BCCAo mice (64). Additionally, CCR5 knockdown in the pre-motor and motor cortices, along with pharmacological inhibition of CCR5, resulted in significant improvements in motor recovery and cognitive function following a stroke. Stroke results in the disruption of connections between neighboring and interacting brain regions. The potential mechanism by which CCR5 knockdown may promote recovery from brain injury could involve either the preservation of synaptic connections in neighboring cortical regions or the enhancement of new synapse formation after the injury. CCR5 knockdown affects recovery through two primary intracellular signaling pathways: CREB and DLK. These pathways are essential for transmitting injury signals, modulating dendritic spine structure, and facilitating axonal regeneration (62). Pharmacological blockers of CCR5 improve recovery after TBI under a mechanism consistent with CCR5 knockdown (65). Conversely, another investigation indicated that the absence of the CCR5 might offer neuroprotection during brain ischemia and reperfusion injury (13). Mice lacking CCR5 exhibited significantly heightened neuronal damage following TBI. The potential protective role of CCR5 in stroke may stem from its ability to modulate the inflammatory response. Consequently, CCR5 might help mitigate brain damage by reducing microglial activation and neuroinflammation.

Vascular dementia is a widespread cause of dementia, second only to AD, attributing to approximately 15% of cases (66). Often coexisting with AD, a combination of vascular and neurodegenerative factors has been identified as a primary contributor to age-related cognitive decline (67). Tournier et al. have presented findings linking the presence of the inactive human form CCR5-Δ32 (in conjunction with ApoEε4) to a heightened risk of dementia (10), particularly in cases of vascular and mixed dementia. Their in-depth study, conducted on mice neurons, shed light on potential mechanisms that contribute to the onset of dementia (10). The research highlighted that oxidative stress triggers an upsurge in neuronal CCR5 expression. Interestingly, the absence of CCR5 in genetically modified mice resulted in neuronal death. This indicates that CCR5 may exert a protective effect on neurons under conditions of vascular damage-induced oxidative stress. Consequently, an increase in CCR5 expression in neurons could be viewed as a defense mechanism, whereas the lack of CCR5 may render neurons more susceptible to apoptosis, potentially heightening the risk of developing vascular dementia.

## 6 CCR5 in multiple sclerosis (MS)

Multiple sclerosis (MS) is a condition marked by inflammation and the loss of myelin within the CNS. Its clinical manifestations are varied, encompassing symptoms such as weakness in the limbs, blurred vision, coordination difficulties, abnormal sensations, fatigue, and cognitive impairments (68). The prevalence of MS ranges from 30/100,000 to 40/100,000 (69).

Analysis of samples from MS patients (70–72) and rodent models of experimental autoimmune encephalomyelitis (EAE) (73, 74) reveals an upregulation of CCR5 in inflammatory brain lesions (Supplementary Table 4). During the progressive phase of the disease, CCR5 appears to be primarily located in infiltrating lymphocytes, macrophages, and microglia within actively demyelinating regions (75). In some cases, CCR5 has occasionally been observed in astrocytes (70) and dendritic cells (76).

In a murine model of MS, specifically EAE, the loss of CCR5 was shown to reduce the severity of demyelination (77). Mice were intraperitoneally administered various doses of maraviroc when early clinical signs of EAE emerged. Administering maraviroc led to a notable reduction in clinical scores and enhanced motor functions. Additionally, maraviroc treatment notably reduced inflammatory cell infiltration into the spinal cord, microgliosis, astrogliosis, levels of pro-inflammatory cytokines, and cell death (78). Furthermore, research by Alghibiwi et al. demonstrated that DAPTA has a significant neuroprotective effect in the EAE model (79), which is mediated by down-regulating inflammatory mediators and affecting the NF-κB/Notch signaling pathway. Collectively, these findings suggest that both maraviroc and DAPTA have potential as therapeutic agents for multiple sclerosis by modulating inflammation and neuroprotection.

## 7 CCR5 in frontotemporal dementia (FTD), dementia with Lewy bodies (DLB), and Parkinson’s disease with dementia (PDD)

Frontotemporal dementia (FTD) is frequently ranked as the second or third most common dementia subtype (80, 81). Torres et al. provided clear evidence of decreased expression of CCL3 and CCR5 in the lymphocytes and monocytes of FTD patients, in contrast to those with AD (11). Higher expression of CCL3 and CCR5 in peripheral cells and its transmigration through the CNS parenchyma might be important, which suggests that peripheral CCL3 and CCR5 expressing cells seem to play a limited role in the pathogenesis of FTD. Certainly, this inference also needs to be investigated more in the future.

*α*-Synuclein (αSyn) is widely recognized for its role in Parkinson’s disease (PD) and is also associated with other synucleinopathies (82, 83), particularly Lewy Body dementias (LBD), which include Dementia with Lewy Bodies (DLB) and PD with dementia (PDD). The accumulation of αSyn in the hippocampus is a key factor in cognitive deficits observed in LBD. Silva et al. showed that elevated αSyn levels lead to cofilin pathology and dendritic spine disruption (12). This process involves a molecular mechanism associated with both cellular prion protein and CCR5. Using an animal model of aSyn, their study also showed that αsyn-induced hippocampal cofilin pathology was associated with synaptic dysfunction and cognitive impairment *in vivo*. Notably, blocking CCR5 with a peptide antagonist fully restored the integrity of dendritic spines in hippocampal neurons affected by αSyn accumulation, which shows promise in future preclinical trials.

## 8 Discussion and prospects

Initially, CCR5 was regarded solely as part of the immune-inflammatory system, primarily involved in leukocyte movement and pathogen defense during infections. However, its biological functions are now recognized to extend beyond this scope. Research has indicated that CCR5 may negatively influence memory circuits and synaptic plasticity (16). Activation of CCR5 can impact neuronal function by inhibiting CREB or MAPK, reducing synaptic plasticity, and hindering axonal regeneration following neuronal injury (16, 62). Although microglia are the principal cells expressing CCR5 in the CNS, the specifics of CCR5’s role in neuron-glial interactions during learning remain uncertain. CCL3/4/5 produced by microglia might significantly affect CCR5 function and, consequently, memory cognition (21, 59). While the majority of studies suggest that CCR5 has a detrimental effect on cognitive functions, there are exceptions, such as in AD and stroke research, where CCR5 loss in mice has been associated with memory impairment.

No significant association was found between the CCR5-delta32 polymorphism and dementia (84). However, it is important to highlight that the statistical power of some studies was limited due to small sample sizes, suggesting that larger cohorts are needed to reliably assess the impact of CCR5-delta32 on dementia. Additionally, population stratification could act as a confounding factor in association studies, as ethnic differences or other variables might influence the frequencies of marker alleles within populations.

Altogether, the studies suggest that CCR5 could indeed influence cognitive function in dementia beyond the well-studied context of HIV, opening up the potential for CCR5-targeted therapies. While this review focuses on CCR5, it’s important to note that other chemokine receptors also impact cognition. Given that CCR5 and similar receptors share ligands and signaling pathways, a deeper understanding of their roles in learning and memory could pave the way for effective treatments for cognitive impairments in various conditions like HAND and AD.
