# Supplementary material for: The contributing role of CCR5 in dementia
Source: Front Neurol. 2025 Jul 9;16:1545302. doi: 10.3389/fneur.2025.1545302 (PMC12283282; doi:10.3389/fneur.2025.1545302)
Supplement: Supplementary file 1 [file Table_1.DOCX]

Supplementary Material

# Supplementary Tables

**Supplementary Table 1**

Studies of CCR5 in HIV-Associated Neurocognitive Disorder (HAND).

| Methods | Model | Main findings | Ref. |
| --- | --- | --- | --- |
| Neuronal survival after 72-h HIV-1 Tat± morphine treatment was tracked in neuronal and glial co-cultures using CCR5-deficient and wild-type mice exposed to the CCR5 antagonist maraviroc or cells exogenously supplemented with BDNF. | Mouse | Loss of glial CCR5 reversed the neurotoxic effects of HIV-1 Tat and morphine.  Maraviroc treatment promotes neuron survival seen in cocultures with CCR5-deficient glia. | [46] |
| Wild-type, Ccr5+/-, and Ccr5-/ -mice were injected with V3 peptide 30 min before training. | Mouse | Knockout or knockdown of CCR5 in the hippocampus effectively prevented memory impairments caused by the HIV V3 peptide.  CCR5 knockout prevented gp120 V3 peptide-induced LTP deficits both in the hippocampus and in the barrel cortex. | [16] |
| Genome-wide gene expression analyses were performed on the brains of HIVgp120tg mice expressing or lacking CCR5 and non-transgenic controls.  Neuronal and behavioral damage were detected in CCR5 knockout HIVgp120 transgenic mice. | Mouse | CCR5 deficiency prevents neuronal damage and reduces microglial activation in gp120-transgenic mice.  Ccr5 knockout (KO) protects spatial learning and memory performance in these HIVgp120tg mice. | [42] |
| Mixed neuronal/glial cultures from rats and mice with one or both of the genetically defective HIV coreceptors were used to determine whether CCR5, CXCR4, or both are HIV/gp120 neurotoxic. | Mouse&Rat | Envelope protein gp120 from CCR5-preferring HIV-1 strains induced neuronal death.  CCL4 or CCL5 protected neurons against neurotoxicity of HIV-1/gp120. | [37] |
| The expression of TNF-α, IL-1β, and IL-6 in primary cultures of rat astrocytes treated with gp120 or cytokines was detected. | Rat | CCR5 activation by CCL3 induced the secretion of TNF-α, IL-1 β, and IL-6.  HIV-1 gp120 activated CCR5 to mediate the secretion of TNF-α, IL-1 β, and IL-6 by astrocytes. | [36] |
| NOD/SCID–IL-2Rγcnull mice, which were engrafted with human blood leukocytes, were either infected with HIV-1, left untreated, or administered maraviroc treatment. | Mouse | Maraviroc notably decreased HIV-induced amyloidogenesis, GSAP levels, phospho-Tau, neurodegeneration, blood-brain barrier (BBB) alterations, and leukocyte infiltration into the CNS.  Targeting CCR5 therapeutically may help reduce viremia, protect the BBB and neurons, and promote brain Aβ clearance. | [47] |
| Assessing maraviroc in the CNS when added to darunavir/ritonavir monotherapy in virologically suppressed HIV-infected subjects. | Human | Possible improvement in executive function. | [33] |
| To evaluate changes in neuropsychological (NP) performance after treatment with cenicriviroc. | Human | Cenicriviroc given over 24 weeks was associated with improved NP test performance. | [34] |
| To investigate whether intensification of combined antiretroviral therapy with the CCR5 entry inhibitor maraviroc improves neurocognitive functioning in men with HAND. | Human | Clinically relevant neurocognitive improvement in HAND patients under maraviroc. | [35] |

**Supplementary Table 2**

Studies of CCR5 in Alzheimer’s Disease (AD).

| Methods | Model | Main findings | Ref. |
| --- | --- | --- | --- |
| Autophagy function was assessed after CCR5 pharmacological or genetic inhibition in Tau mouse models. | Mouse | The defective autophagy-mediated degradation of neurotoxic proteins and the dysfunction associated with mouse models of tau pathology can be reversed by deletion of the maraviroc gene or pharmacologic inhibition of CCR5. | [59] |
| The expression of proteins involved in inflammation was compared between APP/PS1 and wild-type mice. | Mouse | Compared with wild-type mice, APP/PS1 mice had lower CCR5 expression and higher CCL3 and CCL4 expression. | [53] |
| CCL3 (-/-) or CCR5 (-/-) deficient mice were used to investigate the role of chemokines in Abeta (1-40) -induced molecular and behavioral changes. | Mouse | CCR5 − / − mice treated with Aβ1-40 showed reduced activation of microglia and astrocytes.  CCR5 gene deletion prevents cognitive impairments and synaptic degeneration after Aβ injection. | [56] |
| The number of activated microglia and astrocytes was significantly detected 14 days after DAPTA subcutaneous injection in rats. | Rat | DAPTA, a CCR5 antagonist of monocyte chemotaxis, reduced activated microglia, astrocytes, and immunoreactive cells.  DAPTA attenuated neuroinflammation associated with AD. | [57] |
| RNA was prepared from pooled samples of cortical human microglia isolated from postmortem cases, and the consequences of microglial activation at low doses of Abeta 1-42 peptide were investigated in a more extensive manner using gene array technology. | Human | The expression of CCL3 and CCL4 was upregulated in microglia isolated from AD patient brains upon Aβ stimulation. | [54] |
| The expression of CCR3, CCR5, and their ligands in normal and AD brains was investigated by immunohistochemistry. | Human | The expression of CCR5 was increased in reactive microglia and CCL4 was increased in reactive astrocytes in AD brain. | [52] |
| Memory dysfunction, inflammatory protein expression, astrocyte activation, β-secretase expression, and Aβ deposition were assessed after LPS injection in CCR5 wild-type or CCR5 knockout mice. | Mouse | CCR5 KO promoted memory impairment in mice induced by LPS.  CCR5 KO increased LPS-induced Aβ deposits and expression of inflammation-related proteins. | [14] |
| Astrocyte activation, Abeta deposition, and memory dysfunction were evaluated in CCR5 knockout mice. | Mouse | Aβ deposition and CCR2 expression increased in CCR5 - / - mice.  CCR5 deficiency activated astrocytes and impaired memory. | [61] |
| Soluble Aβ_1-42_ was injected into the dorsal CA1 region of the hippocampus. | Mouse | Aβ_1-42_ injection resulted in severe memory impairment.  Aβ1-42 injection caused an increase in Ccr5, Ccl3, and Ccl4 in the dorsal hippocampus. | [55] |
| In mice with targeted gene replacement (TR) of murine *APOE* with human *APOE3* or *APOE4.* | Human& Mouse | CCL5 is increased in  *APOE4*  human CSF and brain lysates from  *APOE4*  TR mice.  Neurophysiological behavioral deficits in heterozygous *APOE4* TR mice are normalized by heterozygous knockout of *CCR5*. | [60] |

**Supplementary Table 3**

Studies of CCR5 in stroke and traumatic brain injury (TBI).

| Methods | Model | Main findings | Ref. |
| --- | --- | --- | --- |
| The effect of neuronal CCR5 knockdown in the premotor cortex on motor recovery and cognitive decline was evaluated after stroke.  One clinical cohort of stroke patients was conducted. | Mouse & Human | CCR5 is differentially upregulated in neurons post-stroke.  Knockdown of CCR5 resulted in the maintenance of dendritic spines, the formation of new axonal connections to the premotor cortex, increased activation of CREB/DLK signaling pathways, and enhanced motor recovery along with improved cognitive performance.  In stroke patients, those carrying the CCR5Δ32 mutation exhibited superior neurological function and cognitive recovery. | [62] |
| The improvement of neurological deficit associated with CCR5 deficiency and reduction of cerebral infarction size was evaluated. | Mouse | The neurological deficits of CCR5 knockout mice were improved, showing a decrease in the percentage of necrotic cavity area and the frequency of ischemic neurons. | [64] |
| Motor behavior, infarct size, and histochemical changes were analyzed at various time points after occlusion of the middle cerebral artery in wild-type and CCR5-deficient mice. | Mouse | Ccr5-deficient mice had increased infarct size, neuronal death, and motor deficits after occlusion compared with wild-type mice. | [13] |
| Pharmacological blockers of CCR5 after TBI. | Mouse | Pharmacological blockers of CCR5 improved recovery after TBI. | [65] |

**Supplementary Table 4**

Studies of CCR5 in multiple sclerosis (MS).

| Methods | Model | Main findings | Ref. |
| --- | --- | --- | --- |
| The effect of DAPTA, a selective CCR5 antagonist, on the secretion of inflammatory mediators and neuroprotection in a mouse model of MS was assessed. | Mouse | DAPTA, a selective CCR5 antagonist, has prominent neuroprotective potential in EAE by down-regulating inflammatory factors and NF-κB/Notch signaling. | [77] |
| The effect of CCR5 antagonist Maraviroc on spinal cord neuroinflammation induced by EAE in mice was examined. | Mouse | Maraviroc treatment led to a marked improvement in behavioral motor functions.  Maraviroc treatment significantly attenuated inflammatory cell infiltration, microgliosis, astrogliosis, proinflammatory cytokines, and cell death in the spinal cord of EAE mice. | [76] |
| An experimental autoimmune encephalomyelitis (EAE) mouse model was used to detect the levels of inflammatory infiltration and cytokine secretion after CCR5 ablation. | Mouse | Clinical scoring and EAE neuropathology were lower in CCR5-/- mice than in CCR5+/+ mice.  Immune cells (e.g., NK cells, and macrophages) infiltration and astrocytes/microglial activation were attenuated in CCR5-/- mice. | [75] |
| In situ hybridization histochemistry was used to examine the local production of CCL3 and CCL5, as well as the cellular presence of CCR5 within inflammatory brain lesions. | Rat | CCL3, CCL5, and large numbers of CCR5 expressing cells showed in inflammatory brain lesions of MS-like rat model. | [72] |
| The effect of γdelta T cell depletion on the expression of chemokines and chemokine receptors was analyzed. | Mouse | CCR5 was elevated in EAE animals at the height of disease in gamma delta T cell-depleted mice.  Exhaustion of gamma delta T cells reduced expression of CCL3 and CCR5 at disease onset, which reduced the number of leukocytes that infiltrated into the CNS. | [71] |
| The migration of peripheral T cells to a panel of chemokines was examined in patients with multiple sclerosis and healthy individuals using Boyden chemotaxis turnwells. | Human | The abnormal migration of MS-derived T cells toward CCL3 and CCL5 is due to overexpression of their receptor, CCR5, and can be blocked by anti-CCR5 antibodies. | [70] |
| Immunostaining was performed to analyze T cell subsets in MS patients with active disease and healthy controls, as well as their expression of IL-2, IFN-γ, and TNF-α. | Human | The number of CCR5+ IFN-γ- and TNF-α producing T cells was increased in the peripheral blood of MS patients. | [69] |
| The expression of CCR2, CCR3, and CCR5 in postmortem MS CNS tissues was examined using single- and double-labeled immunocytochemical techniques. | Human | CCR5 is elevated in the CNS of MS. | [68] |
